# High throughput development of TCR-mimic antibody that targets survivin-2B_80-88_/HLA-A*A24 and its application in a bispecific T-cell engager

**DOI:** 10.1038/s41598-019-46198-5

**Published:** 2019-07-08

**Authors:** Nobuyuki Kurosawa, Yuka Wakata, Kenta Ida, Aki Midorikawa, Masaharu Isobe

**Affiliations:** 1 Laboratory of Molecular and Cellular Biology, Faculty of Science and Engineering, Graduate School, University of Toyama, 3190 Gofuku, Toyama-shi, Toyama, 930-8555 Japan; 2 Frontier Research Core for Life Sciences, University of Toyama, 3190 Gofuku, Toyama-shi, Toyama, 930-8555 Japan; 3 Graduate School of Science and Engineering for Education, University of Toyama, Toyama-shi, Toyama, 930-8555 Japan

**Keywords:** Antibody generation, Tumour immunology

## Abstract

Intracellular tumor-associated antigens are targeted by antibodies known as T-cell receptor mimic antibodies (TCRm-Abs), which recognize T-cell epitopes with better stabilities and higher affinities than T-cell receptors. However, TCRm-Abs have been proven difficult to produce using conventional techniques. Here, we developed TCRm-Abs that recognize the survivin-2B-derived nonamer peptide, AYACNTSTL (SV2B_80-88_), presented on HLA-A*24 (SV2B_80-88_/HLA-A*24) from immunized mice by using a fluorescence-activated cell sorting-based antigen-specific plasma cells isolation method combined with a high-throughput single-cell-based immunoglobulin-gene-cloning technology. This approach yielded a remarkable efficiency in generating candidate antibody clones that recognize SV2B_80-88_/HLA-A*24. The screening of the antibody clones for their affinity and ability to bind key amino-acid residues within the target peptide revealed that one clone, #21-3, specifically recognized SV2B_80-88_/HLA-A*24 on T2 cells. The specificity of #21-3 was further established through survivin-2B-positive tumor cell lines that exogenously or endogenously express HLA-A*24. A bispecific T-cell engager comprised of #21-3 and anti-CD3 showed specific cytotoxicity towards cells bearing SV2B_80-88_/HLA-A*24 by recruiting and activating T-cells *in vitro*. The efficient development of TCRm-Ab overcomes the limitations that hamper antibody-based immunotherapeutic approaches and enables the targeting of intracellular tumor-associated antigens.

## Introduction

Antibody therapy has targeted tumor-specific and tumor-associated antigens (TAA) expressed on the cell surface. However, the majority of such TAA are localized within the cells; therefore, they have not been pursued for antibody therapies. Intracellular proteins are proteolytically processed to 8- to 11-amino acid fragments in the cytosol by the proteasome. The peptides are loaded on the groove of major histocompatibility complex class I molecules (also called human leukocyte antigen, HLA) and presented on the cell surface as peptide/HLA complexes, which enables recognition by T-cell receptors (TCRs) on T-cells^1^. However, the use of TCRs as therapeutic agents has been hindered by their inherent low affinity and their instability as recombinant molecules^2,3^. To this end, antibodies known as T-cell receptor mimic antibodies (TCRm-Abs), which recognize epitopes similar to those recognized by TCRs, have been developed^4–6^. However, it has been proven difficult to generate high-affinity and high-specificity TCRm-Abs by either display or hybridoma approaches because they recognize conformational epitopes consisting of an invariant HLA molecule with a variable short linear peptide embedded within it^5^.

Survivin is a tumor-associated antigen that is highly expressed in tumors, and it plays an important role in tumor cell proliferation, apoptosis, invasion and metastasis^7^. A splicing variant of survivin, survivin-2B, was also highly expressed in a wide variety of cancer cells but was low or non-existent in normal cells. Furthermore, survivin-2B contains the epitope peptide, AYACNTSTL (SV2B_80-88_), which is capable of binding to HLA-A*24. Moreover, the SV2B_80-88_ could induce a cytotoxic T lymphocytes (CTL) response in the context of HLA-A*24, and the CTL showed cytotoxicity against tumor cells expressing survivin-2B^8,9^. As HLA-A*24 is a very frequently expressed allele, especially in the Asian population, we selected the SV2B_80-88_ as a target peptide for the production of TCRm-Abs that recognize SV2B_80-88_ presented on HLA-A*24 (SV2B_80-88_/HLA-A*24).

We recently developed a fluorescence-activated cell sorting (FACS)-based antigen-specific plasma cells (PCs) isolation method, which we termed (ERIAA), for rapid and scalable automation in monoclonal antibody (mAb) generation^10^. ERIAA is based on features of PCs, abundant cytoplasmic rough endoplasmic reticulum (ER) that can be used for PC identification with ER-specific florescent dye (ER-tracker) and weakly expressed cell surface IgG that can be used as a tag for a complementary fluorescently labeled antigen. By applying ERIAA with fluorescently labeled HLA-tetramers and a high-throughput single-cell-based immunoglobulin-gene-cloning technology, we succeeded to generate mAb clones that bind to the SV2B_80-88_/HLA-A*24 from SV2B_80-88_/HLA-A*24-immunized mice. The mAb clones were analyzed for their affinity and ability to bind key amino acid residues within the SV2B_80-88_, which revealed that one mAb clone (#21-3) showed the highest binding specificity with the equilibrium dissociation constant (K_D_) = 7.4 nM. A bispecific T-cell engager (BiTE) comprised of #21-3 and CD3 showed specific cytotoxicity towards cells bearing the SV2B_80-88_/HLA-A*24 *in vitro* by crosslinking T-cells to the target cells. To the best of our knowledge, this is the first TCRm-Ab that targets the antigenic peptide derived from intracellular survivin-2B in the context of HLA-A*24.

## Results

### Isolation of candidate TCRm-Abs that target SV2B_80-88_/HLA-A*24

**Figure 1:**
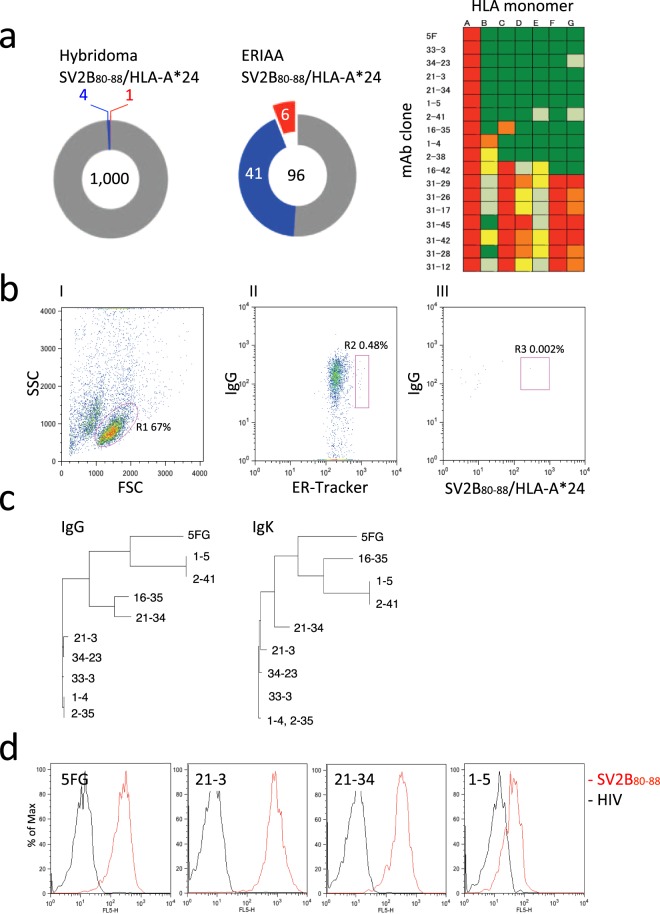
Development of monoclonal antibodies against SV2B_80-88_/HLA-A*24. (**a**) Characterization of binding specificity of mAbs by ELISA with HLA-A*24 monomers. Crude mAbs obtained from hybridoma or ERIAA were used to probe wells coated with HLA-A*24 monomers loaded on different peptides. Pie charts represent antibody binding patterns, colour-coded as follows: mAb clones that reacted with only SV2B_80-88_/HLA-A*24 monomer (red), mAb clones that reacted with SV2B_80-88_/HLA-A*24 and irrelevant HLA-A*24 monomers (blue), mAb clones that did not react with SV2B_80-88_/HLA-A*24 monomer (grey). The number in the centre of the pie denotes the number of mAb clones screened. A coloured heat map (right) shows the relative immunoreactivity of each mAb clone against SV2B_80-88_/HLA-A*24 monomer compared to that against irrelevant peptide/HLA-A*24 monomers. A: SV2B_80-88_, B: HIVgp160, C: NY-ESO, D: SOX2-1, E: SOX2-2, F: MAGE3A-1 and G: MAGE3A-2. Signal intensities are colour-coded as follows: light green (<0%), green (>0–25%), yellow (>25–50), >50–75% (orange) and >75% (red). Values are represented as the means of two replicates. (**b**) FACS gating strategy for the isolation of SV2B_80-88_/HLA-A*24-specific PCs by ERIAA. Splenocytes were stained with anti-mouse IgG, ER-tracker and SV2B_80-88_/HLA-A*24-tetramer. Plots (I)–(III) represent the sequential gating strategy. (I) FSC vs SSC with gate R1 represent lymphocytes. (II) The anti-mouse IgG^Low^ ER-Tracker^High^ fraction was defined as PCs. (R2). (III) The SV2B_80-88_/HLA-A*24-specific PCs were defined as IgG^Medium^ ER-Tracker^High^ SV2B_80-88_/HLA-A*24^High^ (R3 gate). Numbers indicate the percentages of cells in the gated area. 50,000 events were recorded. (**c**) Phylogenetic analysis of V_H_ and V_L_ amino-acid sequences of SV2B_80-88_/HLA-A*24-specific mAb clones. (**d**) FACS analysis of the candidate mAbs by T2/A24 cells. T2/A24 cells (1 × 10^5^) pulsed with either SV2B_80-88_ or HIV were stained with crude candidate mAbs. The binding ability of each mAb was evaluated by mean fluorescent intensity (MFI) of stained T2/A24 cells.

**Figure 2:**
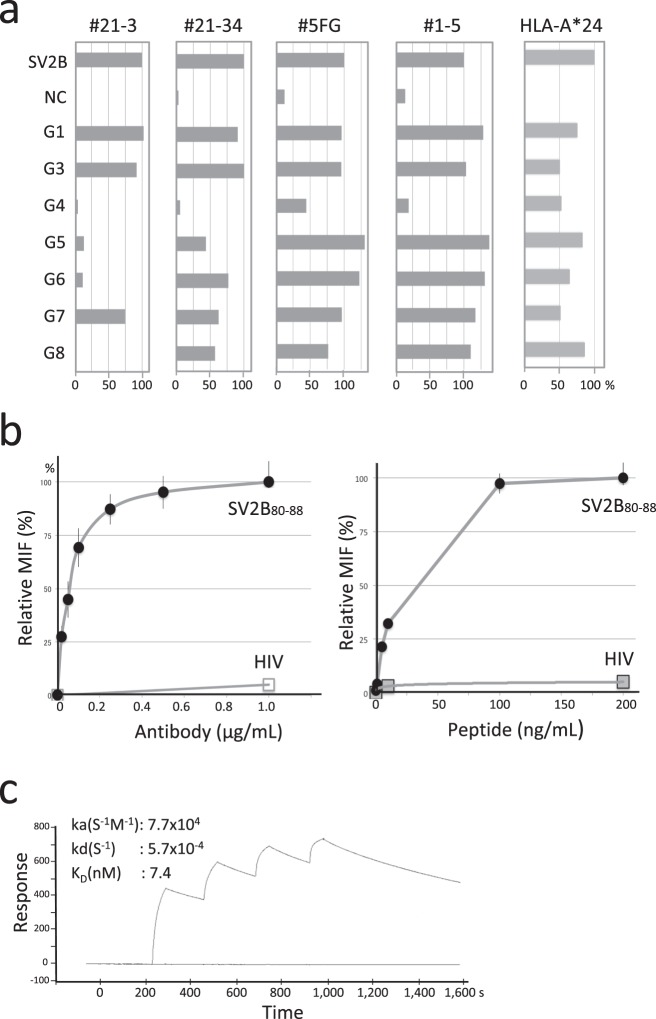
Characterization of binding specificity of #21-3 against SV2B_80-88_/HLA-A*24. (**a**) Epitope mapping of the candidate mAbs based on glycine-substituted SV2B_80-88_ peptides. Each non-anchor residue in the SV2B_80-88_ was substituted for glycine, and peptides were pulsed onto T2/24 cells. Binding of the mAbs was determined by FACS relative to native SV2B_80-88_-pulsed T2/A24 cells. T2/A24 cells (1 × 10^5^) pulsed with either SV2B_80-88_ or glycine-substituted SV2B_80-88_ peptides (0.2 µg/mL) are incubated with the selected mAbs (0.1 µg/mL) and the PE-labeled anti-HLA-A*24 mAb. The antibody binding was evaluated by MFI of the stained T2/A24 cells. NC, T2 cells without peptide. Values are represented by the means of two independent experiments for two replicates/group. (**b**) Binding of #21-3 to SV2B_80-88_/HAL-A*24 on T2/A24 cells. T2/A24 cells pulsed with the SV2B_80-88_ or HIV at 0.2 µg/mL were stained with #21-3 at the indicated concentrations (upper panel). T2/A24 cells pulsed with the SV2B_80-88_ at various concentrations were stained with #21-3 at 1.0 μg/mL. HIV pulsing at 0.2 µg/mL was used as a control (lower panel). Relative MFI of the staining were plotted. Data in each panel is representative of two independent experiments (n = 3). (**c**) SPR analysis of #21-3 for affinity to SV2B_80-88_/HLA-A*24. Calculated affinity constants are displayed. Data are representative of two independent experiments.

First, we attempted to develop TCRm-Abs by conventional B-cell hybridoma from splenocytes of mice that were immunized with SV2B_80-88_/HLA-A*24 as an antigen. Upon screening of 1,000 hybridoma clones by ELISA, five clones showed a positive signal with the SV2B_80-88_/HLA-A*24 monomer. Additional ELISA screening of these clones was conducted with a panel of irrelevant peptide/HLA-A*24 monomers, HIVgp160 (HIV), NY-ESO, SOX2-1, SOX2-2, MAGE3A-1 and MAGE3A-2; this analysis revealed that only one clone (5FG) showed the required specificity for SV2B_80-88_/HLA-A*24 (Fig. 1a). The low probability of obtaining a candidate TCRm-Ab by hybridoma prompted us to use our recently developed ERIAA and high-throughput single-cell-based immunoglobulin-gene-cloning technology. The splenocytes from the immunized mice were stained with anti-mouse IgG, ER-tracker and SV2B_80-88_/HLA-A*24 tetramer; the SV2B_80-88_/HLA-A*24-specific PCs gated as IgG^Medium^ ER-tracker^High^ and SV2B_80-88_/HLA-A*24^High^ (R3 gate) were single-sorted by FACS (Fig. 1b). Single cell-based immunoglobulin heavy chain variable (V_H_) and light chain variable (V_L_) gene amplification was conducted by PCR of the R3-gated cells, followed by the DNA transfection of cognate pairs of immunoglobulin heavy and light chain gene into 293FT-cells, which resulted in the production of recombinant mAbs. The screening of 96 clones by ELISA with a panel of peptide/HLA-A*24 monomers revealed that 47 clones bound to the SV2B_80-88_/HLA-A*24 monomer, among which six clones (#33-3, #34-23, #21-3, #21-34, #1-5 and #2-41) did not bind to six irrelevant peptide/HLA-A*24 monomers (Fig. 1a). The DNA sequencing revealed that these mAb clones were divided into three phylogenetic clusters (Fig. 1c). We selected four mAb clones from each cluster (5FG, 21-3, 21-34 and 1-5) and analyzed their specificity with T2 cells stably expressing HLA-A*24 (T2/A24). As shown in Fig. 1d, all mAbs appeared to bind to SV2B_80-88_-pulsed T2/A24 cells but not to HIV-pulsed cells. To map key amino-acid residues that are involved in antibody interactions, each residue on the SV2B_80-88_ was replaced with glycine (except the canonical anchor residues on positions 2 and 9), and mAb binding was assessed on T2/A24 cells. As shown in Fig. 2a, #21-3 showed the widest epitope coverage; substitutions on positions 4, 5, 6 and 8 abrogated the binding; and position 7 reduced the binding by 52%. However, substitutions on either position 1 or 3 did not abrogate the binding. On #21-34, substitutions on position 4 abrogated the binding and that on any of positions 5, 6, 7 or 8 reduced the binding by 52~75%; however, substitutions on either position 1 or 3 did not abrogate the binding. #5FG and #1-5 were relatively insensitive to substitution at all positions except position 4; their recognition patterns correspond to the phylogenetic data shown in Fig. 1c. Based on the glycine substitution analysis, #21-3, reacting with the C-terminus of SV2B_80-88_, was selected and subjected to further analysis.

**Figure 3:**
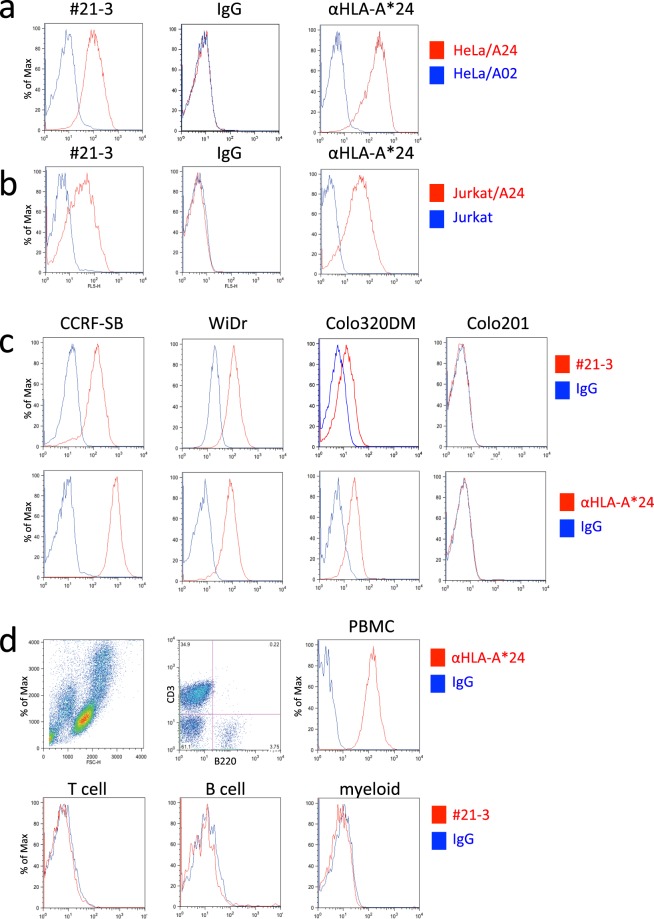
#21-3 detects endogenous SV2B_80-88_/HLA-A*24 expressed on human tumor cell lines. (**a**) HeLa/A02 or HeLa/A24 cells were labeled with #21-3 (0.1 µg/mL) or control mouse IgG for 30 min at 4 °C and were analyzed by FACS. The integrity of the peptide/HLA-A*24 expression was confirmed with an antibody against HLA-A*24. Representative FACS histograms are shown. (**b**) Jurkat/A24 cells were stained with #21-3 or control mouse IgG and were analyzed as described above. (**c**) The indicated human tumor cell lines were stained with #21-3 or control mouse IgG, and binding was determined as described above. (**d**) PBMC from HLA-A*24^+^ healthy donors were stained with #21-3. A representative gating strategy and #21-3 histogram compared with control mouse IgG are shown. All data are representative of two independent experiments.

The dose-response of #21-3 was tested with respect to its binding to the SV2B_80-88_/HLA-A*24 on T2/A24 cells. T2/A24 cells pulsed with SV2B_80-88_ showed increased #21-3 binding when the antibody concentration was increased, and the binding was saturated at 1.0 μg/mL. Likewise, increasing SV2B_80-88_ concentrations also increased #21-3 binding, which indicated that the antibody binding is dependent on the density of the SV2B_80-88_/HLA-A*24 on T2/A24 cells (Fig. 2b). The antibody binding affinity and kinetics were determined by surface plasmon resonance (SPR). As shown in Fig. 2c, the binding kinetics was characterized by a fast association rate (k_a_ = 7.7 × 10^4^ M^−1^ s^−1^) and a slow dissociation (k_d_ = 5.7 × 10^−4^ s^−1^) with high affinity (K_D_ = 7.4 nM). To establish the ability of #21-3 to detect endogenously processed SV2B_80-88_ in the context of HLA-A*24, HeLa cells (HLA-A*68^+^, survivin-2B^+^) and Jurka cells (HLA-A*03^+^, survivin-2B^+^) were transfected with a plasmid expressing HLA-A*24 to generate a stable transformant (HeLa/A24 and Jurkat/A24); the binding of #21-3 to those cells were examined by FACS. As shown in Fig. 3a,b, specific binding was only observed on HeLa/A24 and Jurkat/A24, but neither on HeLa cells stably expressing HLA-A*02:01 (HeLa/A02) nor on parent Jurkat cells, respectively. We also investigated whether #21-3 could detect naturally processed SV2B_80-88_/HLA-A*24 by using a panel of tumor cell lines. As shown in Fig. 3c, #21-3 bound to HLA-A*24^+^ survivin-2B^+^ cell lines (CCRF-SB, WiDr and Colo320DM), but not to HLA-A*24^−^ survivin-2B^+^ cell line (Colo201). We detected no #21-3 binding on T, B, and myeloid populations in peripheral blood mononuclear cells (PBMC) that were collected from a HLA-A*24^+^ healthy donor (Fig. 3d). These results indicate that #21-3 specifically binds to SV2B_80-88_/HLA-A*24 but does not react with other cell surface antigens expressed on T2, Hela, Jurkat, Colo201 and PBMCs. Overall, the specificity, affinity, and kinetics of binding suggest that #21-3 possesses characteristics that are required for a TCRm-Ab.

### #21-3BiTE directs T-cells to kill human tumor cell lines *in vitro*

To establish the ability of the #21-3 to inhibit tumor growth *in vitro*, we generated a BiTE by fusing an anti-CD3 single-chain variable fragment (scFv) to the C terminal of the full length of #21-3 (#21-3BiTE) (Fig. S1a,b). To ascertain that #21-3BiTE binds simultaneously to CD3 and SV2B_80-88_/HLA-A*24, PBMC from a HLA-A*24^−^ healthy donor and WiDr cells were stained with #21-3BiTE. The anti CD3 ScFv arm of #21-3BiTE stained CD4^+^ and CD8^+^ T-cells, but the binding was 10-fold lower than that of the parent anti-CD3 antibody (OKT3) (Fig. S1c,d). #21-3BiTE retained a similar binding specificity to WiDr cells as the parent antibody (Fig. S1e). These results demonstrate that #21-3BiTE contains the antigen-binding capacity of each of the parent antibodies.

**Figure 4:**
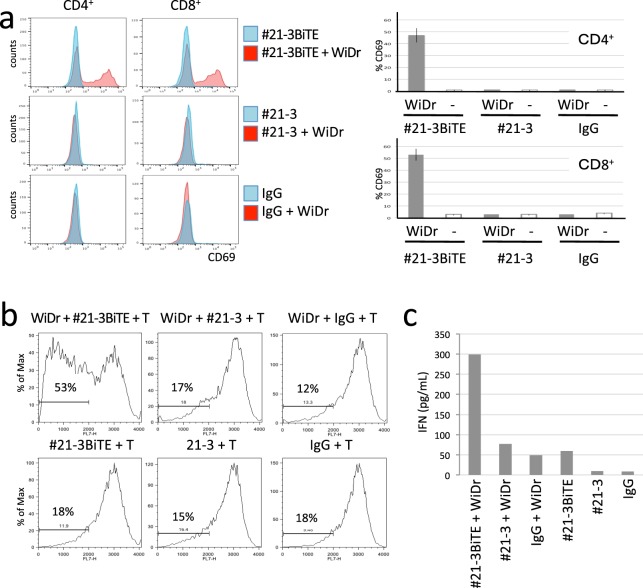
#21-3BiTE mediates T-cell activation and proliferation. (**a**) #21-3BiTE activates naïve T-cells by bridging T cells and target cells. PBMC from HLA-A*24^+^ healthy donor were mixed with or without WiDr cells at an E:T ratio of 10:1 in the presence of #21-3BiTE, 21-3 or control mouse IgG, and the reactions were allowed to proceed for 48 h. Cells were labeled with fluorescently coupled antibodies to CD4, CD8, and CD69 and were analyzed by FACS. Each FACS histogram shows CD69 binding. Representative FACS histograms (left) and frequency of CD69-positive cells in CD4^+^ or CD8^+^ cells are shown (Right). Data are representative of two independent experiments (n = 3). (**b**) T cells expanded from a HLA-A*24^+^ healthy donor were labeled with CellTrace-Blue. Labeled T-cells were mixed with or without WiDr cells at an E:T ratio of 10:1 in the presence of the indicated antibody, and the reactions were allowed to proceed for four days. CellTrace-Blue^+^ cells analyzed by FACS for proliferation are shown. Representative data from two independent experiments are shown. (**c**) Cell culture supernatants in (**b**) were analyzed for IFNγ by ELISA. Values are represented by the means of two independent experiments for two replicates/group.

To assess if #21-3BiTE activates naïve T-cells by bridging T-cells and target cells, PBMC from a HLA-A*24^+^ healthy donor were cultured with #21-3BiTE, #21-3 or control mouse IgG in the presence or absence of WiDr cells. T-cell activation was measured by assessing the expression of the activation marker CD69. Upregulated CD69 expression on both CD4^+^ and CD8^+^ T-cells was observed only when PBMC were incubated with #21-3BiTE and WiDr cells (Fig. 4a). To determine if the BiTE drives T-cell proliferation, CellTrace-Blue-stained T-cells expanded from a HLA-A*24^+^ healthy donor were cultured with #21-3BiTE, #21-3 or control mouse IgG in the presence or absence of WiDr for four days. Approximately 50% of the T-cells proliferated in the presence of #21-3BiTE and the tumor, whereas T-cells cultured with or without WiDr cells in the presence of #21-3 or control mouse IgG did not show potent proliferation (Fig. 4b). When T-cell activation was further evaluated based on secretion of the interferon (IFN)-γ, only T-cells incubated with WiDr cells in the presence of #21-3BiTE showed an elevated expression of IFN-γ. T-cells incubated without WiDr cells in the presence of #21-3BiTE secreted low level of IFN-γ, which was probably due to the non-specific T-cell activation mediated through the anti CD3 arm of #21-3BiTE (Fig. 4c).

**Figure 5:**
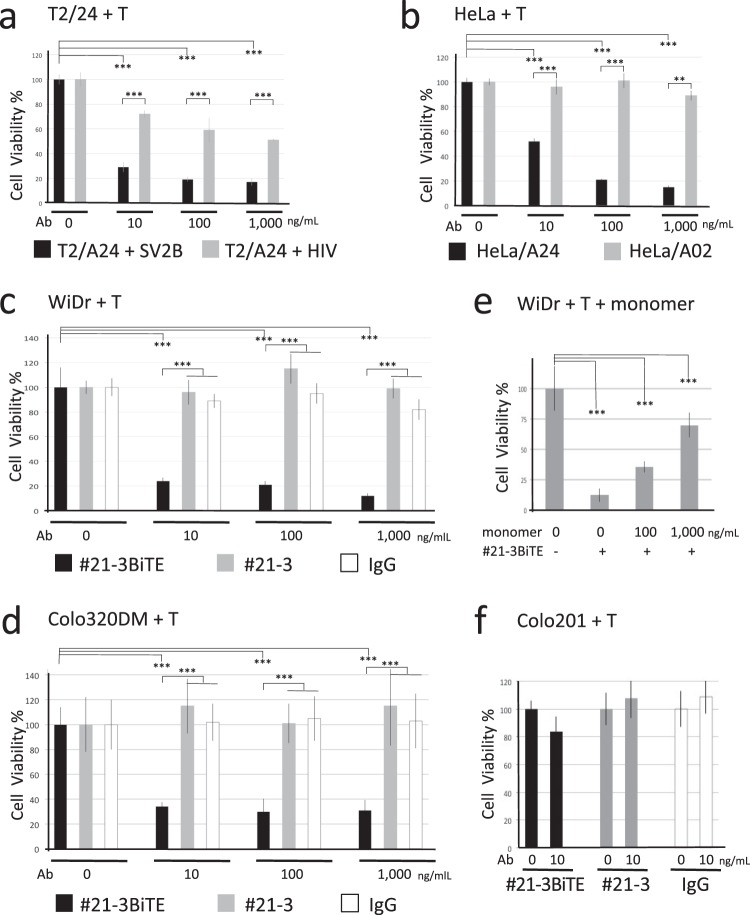
#21-3BiTE shows potent T-cell dependent cytotoxicity against surviving-2B^+^ HLA-A*24^+^ tumor cells *in vitro*. (**a**) T2/A24 cells stably expressing luciferase were pulsed with the indicated peptide and then mixed with T-cells expanded from a HLA-A*24^+^ healthy donor at an E:T ratio of 10:1 in the presence of serial dilutions of #21-3BiTE. The reactions were allowed to proceed for 8 h. Target cell viability was determined by luciferase assay. Data are representative of two independent experiments. (**b**) HeLa/A24 or HeLa/A02 cells stably expressing luciferase were mixed with T-cells at E:T ratio of 10:1 in the presence of serial dilution of #21-3BiTE, and the reactions were allowed to proceed for 12 h. Relative cell viability values for HeLA cells is determined by luciferase assay. Data are representative of three independent experiments. (**c**) WiDr cells stably expressing luciferase were mixed with the T-cells at an E:T ratio of 10:1 in the presence of serial dilution of the indicated antibody, and the reactions were allowed to proceed for 14 h. Relative cell viability values for WiDr cells was determined by luciferase assay. Representative data from three independent experiments are shown. (**d**) Colo320DM cells were treated as in (**c**), and relative cell viability values was determined by luciferase assay. (**e**) WiDr cells mixed with the T-cells at an E:T ratio of 10:1 were incubated with 10 ng/mL of #21-3BiTE in the presence of serial dilutions of SV2B_80-88_/HLA-A*24 monomer, and the reactions were allowed to proceed for 14 h. Relative cell viability values for WiDr cells was determined by luciferase assay. Data are representative of two independent experiments. (**f**) Colo201 cells were treated as in (**c**), and the reactions were allowed to proceed for 14 h. Target cell viability was determined by luciferase assay. Relative cell viability values for target cells are represented as the average ± SD for three replicates/group (***p < 0.001).

Next, the ability of #21-3BiTE to crosslink T-cells to target cells was analyzed. Incubation of SV2B_80-88_-pulsed T2/A24 cells with T-cells expanded from a HLA-A*24^+^ healthy donor in the presence of #21-3BiTE induced potent T-cell cytotoxicity in a dose-dependent manner. In contrast, T-cell-mediated cytotoxicity of HIV-pulsed T/A24 cells by #21-3BiTE was relatively ineffective (Fig. 5a). #21-3BiTE also showed specific cytotoxicity against HeLa/A24 cells in co-cultures of the T-cells (Fig. 5b). Next, we tested the therapeutic efficacy of #21-3BiTE by using cell lines endogenously expressing both surviving-2B and HLA-A*24. WiDr and Colo320DM cells stably expressing luciferase were mixed with the T-cells expanded from a HLA-A*24^+^ healthy donor in the presence of #21-3BiTE, #21-3 or control mouse IgG, and cytotoxicity was measured. As shown in Fig. 5c,d, specific cytotoxicity was only observed on #21-3BiTE but neither on #21-3 nor on control mouse IgG. #21-3BiTE-induced T-cell cytotoxicity against WiDr cells was inhibited by the addition of the SV2B_80-88_/HLA-A*24 monomer in a dose-dependent manner (Fig. 5e). The BiTE-dependent T-cell-mediated cytotoxicity of Colo201 cells was relatively ineffective (Fig. 5f). Similar results were also observed when using T-cells expanded from a HLA-A*24^−^ donor (Fig. S2a–d). These results indicate that reduced tumor cell viability is induced by specific binding of T-cells to SV2B_80-88_/HLA-A*24 on tumor cells through #21-3BiTE, supporting the conclusion that #21-3BiTE has a potential for therapeutically targeting tumor cells that express SV2B_80-88_/HLA-A*24.

## Discussion

Therapeutic antibodies are one of the most successful biological drugs used to treat cancers. However, their application has been limited to extracellular or cell-surface proteins because antibodies are too large to cross the cell membrane to target intracellular proteins. TCRm-Abs can recognize intracellular antigens in the form of peptides loaded on the HLA, thereby increasing the opportunity to expand the repertoire of therapeutic antibodies. Several groups have attempted to generate TCRm-Abs by conventional hybridoma techniques; however, these attempts have shown a very low probability of success because many antibodies bind epitopes that are not directly involved in the target peptide presented on HLA^11–16^. Our attempts to isolate TCRm-Ab by hybridoma also resulted in low efficiency. Overall, obtaining specific TCRm-Ab has been proven to be difficult by conventional hybridoma techniques. Our FACS-based strategy combined with single-cell immunoglobulin-gene-cloning technology enables to isolate rare antigen-specific PCs, resulting in a remarkable efficiency in generating candidate mAbs that recognize SV2B_80-88_ in complex with HLA-A*24 from antigen-immunized mice. One selected mAb clone, #21-3, was capable of recognizing SV2B_80-88_/HLA-A*24, discriminating normal PBMC from tumor cells. #21-3-derived BiTE selectively mediates anti-tumor reactivity, indicating a potential therapeutic tool capable of targeting malignancies.

TCRm-Abs have been known to recognize off-target epitopes that share homologous peptide sequences^4,17^. Given that #21-3 primarily contacts the C-terminal half of SV2B_80-88_ (positions 4~8), this TCRm-Ab may cross-react with antigenic peptides sharing the key amino acids essential for antibody binding. In addition to the off-target binding, the risk of on-target/off-tumor recognition caused by target antigen expression on normal cells is also a major obstacle for antibody-based therapies^18^. Survivin-2B has been found to be expressed by several stem cells, thereby #21-3 might have off-tumor’s reactivity that target stem cells^19^. We have shown that #21-3 did not bind to PBMC from a healthy HLA-A*24^+^ donor, which suggested there was no broad expression of off-target and off-tumor epitopes on normal cells, at least in differentiated haematopoietic cells.

The significantly low antigenic density of a peptide/HLA on tumor cell surface is another obstacle for TCRm-Ab therapy, which limits the efficacy of TCRm-Abs to stimulate immune effectors for antibody-dependent cytotoxicity. To overcome this problem, BiTEs that bridges TAA on tumor cells and CD3 on T-cells to enhance the cytotoxic activity of T cells against tumor cells have been developed^20–22^. Our novel TCRm-BiTE could bridge T-cells via its anti CD3 ScFv arm and tumor cells via its #21-3 arm, resulting in activation and proliferation of T-cells; this result demonstrated the efficacy of the TCRm-BiTE in targeting the intracellular survivin-2B in the context of HLA-A*24. Further investigations will be needed to evaluate the risks and benefits associated with the #21-3TCRm-Ab to constrain the activity of the TCRm-BiTE to avoid undesirable side effects and maximize efficacy.

## Methods

### Materials

HLA monomers for HLA-A*24; SV2B_80-88_ (AYACNTSTL), glycine substitution variants for each amino-acid position of SV2B_80-88_, HIVgp160 (RYLRDQQLL), NY-ESO (LLMWITQCF), SOX2-1 (KYTLPGGLL), SOX2-2 (KYRPRRKTKTL), MAGE3A-1 (IMPKAGLLI), MAGE3A-2 (TFPDLESEF) and T-Select human MHC Tetramer for SV2B_80-88_**/**HLA-A*24 were purchased from MBL (https://www.mblintl.com). cDNAs encoding HLA-A*24:02 and HLA-A*02:01 were purchased from RIKEN RBC (http://cell.brc.riken.jp/en/). Antibodies specific for HLA-A*02 (BB7.2) and HLA-A*24 (17A10) were obtained from BioLegend (https://www.biolegend.com) and MBL, respectively. Anti-human CD4, CD8 and CD69 were purchased from Miltenyi Biotec (https://www.miltenyibiotec.com). ELISA kits for human IFN-γ was purchased from R&D SYSTEMS (https://www.rndsystems.com). Firefly luciferase substrate BrillianStar-LT was purchased from FUJIFILM (http://ffwk.fujifilm.co.jp).

### Cell culture and stable cancer cell lines

HLA-A*24^+^ and HLA-A*24^−^ human peripheral blood mononuclear cells (PBMC) were purchased from Cellular Technology Limited (www.immunospot.com). T-cells isolated with Pan T-cell Isolation Kit (Miltenyi Biotec https://www.miltenyibiotec.com) were expanded with Dynabeads Human T-Activator CD3/CD28 (Thermo Fisher Scientific, https://www.thermofisher.com) and IL2 in GT-T551 culture medium (Takara Bio, http://www.takara-bio.co.jp) that contained 10% fetal bovine serum (FBS). The T-cells were cultured without adding IL-2 and CD3/CD28 beads for 24~48 h to allow the cells to stop proliferating and enter into a resting mode. Tumor cell lines were purchased from RIKEN RBC, JCRB (http://cellbank.nibiohn.go.jp/english/) and ATCC (https://www.atcc.org). T2 cells (ATCC CRL 1992), HeLa cells (RIKEN RCB0007), Jurka cells (RIKEN RCB3052), Colo320DM (RIKEN RCB1193), WiDr, (JCRB IFO50043), Colo201 (JCRB 0226) and CCRF-SB (JCRB 0032) were cultured in IMDM medium supplemented with 10% FBS. cDNA encoding HLA-A*02:01 or HLA-A*24:02 was sub-cloned into CSII-CMV-MCS-IRES2-Bsd vector (RIKEN RBC). Lentiviral particles were generated by individually transfecting 293FT-cells with lentiviral vector and the ViraPower Packaging Mix (Takara Bio) with Lipofectamine 3000 according to the manufacturer’s protocol (Thermo Fisher Scientific). At 48 h after transfection, the culture supernatants were collected, and lentiviral particles were concentrated by Lenti-X Concentrator, according to manufacturer’s instructions (Takara Bio). T2 cells and HeLa cells were transduced with the lentiviral particles encoding HLA-A*02:01 or HLA-A*24:02 and selected with blasticidin to establish T2/A24, HeLa/A24 and HeLa/A02 cells, repectively. Firefly luciferase gene was sub-cloned into pFC-EFa-MCS-PGK-RFP-Puro and were transfected into T2/A24, HeLa/A24, HeLa/A02, WiDr, Colo320DM and Colo201 cells by using phiC31-integrase-mediated recombination (System Biosciences, https://www.systembio.com). Stable cell lines expressing luciferase were obtained by puromycin selection.

### Immunization

Groups of three mice were immunized at 3-week intervals for a total of 3 times by intraperitoneal injection of SV2B_80-88_/HLA-A*24:02 monomer antigen (50 µg) plus TiterMax adjuvant, followed by an intraperitoneal injection of antigen alone 7 days before harvesting splenocytes.

### Generation of TCRm antibodies

Isolation of TCRm-specific PCs was performed as previously described, with slight modifications. Mouse splenocytes (1 × 10^7^/mL) were suspended in 1 mL of PBS-BSA and stained with PE-labeled SV2B_80-88_/HLA*A24:02-tetramer (0.1 µg/mL) and fluorescently labeled antibody against mouse IgG at 4 °C for 30 min with gentle agitation^23^. After washing with phosphate buffered saline (PBS), the cells were suspended in 4 mL of PBS containing ER-tracker (0.25 µM) and, subsequently, analyzed by FACS. The forward-versus-side-scatter (FSC vs SSC) lymphocyte gate (R1) was applied to exclude dead cells. The PCs (IgG^Medium^ ER-tracker^High^, R2 gate) were further subdivided into fractions according to their binding of fluorescently labeled HLA tetramer to define the antigen-specific PCs (IgG^Medium^ ER-tracker^High^ SV2B_80-88_/HLA-A*24^High^). Single-cell sorting was performed using a JSAN Cell Sorter that was equipped with an automatic cell deposition unit (http://baybio.co.jp/english/top.html) with fluorescently labeled antibody against IgG monitored in the FL-l channel, PE-labeled SV2B_80-88_/HLA-A*24-tetramer in the FL-2 channel and ER-tracker in the FL-7 channel. Molecular cloning of V_H_ and V_L_ genes from single cells and recombinant antibody expression were performed as previously described^24^. Antibodies were produced via the Expi293 cell culture system according to the manufacturer’s protocol (Thermo Fisher Scientific). Antibodies were purified via protein G column chromatography and then analyzed by SDS-PAGE. Hybridoma production was outsourced to TransGenic Inc. (http://www.transgenic.co.jp/en/). Large-scale production of #21-3BiTE was outsourced to Absolute Antibody Ltd. (https://absoluteantibody.com).

### ELISA

Streptavidin-coated 96-well plates (SUMITOMO BAKELITE, https://www.sumibe.co.jp) were coated with 50 ng of biotinylated peptide/HLA-monomers in 50 μl of PBS at 4 °C overnight. Plates were washed with PBS, and 100 µl of crude mAbs were added to each well. After 1 h incubation at room temperature, bound mAbs were detected by alkaline phosphatase-conjugated goat anti-mouse IgG (Sigma) with KPL BluePhos Microwell Phosphatase Substrate System (Roche, https://www.roche.com) and then quantified with a Varioskan LUX multimode reader (Thermo Scientific).

### Antibody affinity and kinetic assay

SPR single-cycle kinetic experiments were performed by an indirect capture method using a Biacore T100 instrument (GE Healthcare, https://www.gelifesciences.com/en/us). Briefly, two adjacent channels on a CM5 sensor chip were immobilized with IgG binder using Mouse Antibody Capture Kit according to the manufacturer’s recommendations. Purified TCRm-Ab was injected at a flow rate of 10 μl/min to achieve a ligand immobilization level of 200 to 400 relative units. Five different concentrations of each of the HLA monomer (ranging from 0 nM to 30 nM) were then injected at a flow rate of 30 μl/min for 1 min, followed by 5 min of washing with HBS-P buffer. The single-cycle kinetic curves were fitted with 1:1 binding stoichiometry for ka, kd and K_D_.

### Detection of peptide/HLA on target cells with FACS

T2/A24 cells (1 × 10^5^ cells) were incubated with peptides at various concentrations overnight at 30 °C in a 96-well tissue culture plate. Following 1 h incubation at 37 °C, the T2/A24 cells were harvested and labeled with purified TCRm-Abs for 30~60 min at 4 °C. Cells were labeled with Goat anti-Mouse IgG (H + L) DyLight® 650 (Thermo Fisher Scientific) for 30 min at 4 °C, washed with PBS. Cell surface expression of HLA-A*24 or HLA-A*02 was determined by direct staining of the cells with the respective mAbs. FACS data were collected on a JSAN Cell Sorter and analyzed with the FlowJo software (BD Biosciences, http://www.bdbiosciences.com/ca/home).

### BiTE-induced T-cell activation and proliferation

PBMC from healthy donors (1 × 10^5^ cells) were mixed with or without WiDr cells (1 × 10^4^ cells) in 100 μl of GT-T551 culture medium and were incubated with #21-3BiTE, #21-3 or mouse IgG in triplicate in a total volume of 200 μl in 96-well flat-bottom plates. Following 2 days of incubation, the cells were stained with fluorescent antibodies against CD4, CD8 and CD69. Gated CD4^+^ and CD8^+^ T-cells were assessed for the expression of CD69 by FACS. T-cells were labeled using a CellTrace-Blue kit according to the manufacturer’s directions (Thermo Fisher Scientific). Labeled T-cells (1 × 10^5^ cells) were mixed with or without WiDr cells (1 × 10^4^ cells) in 96-well plates and cultured in 100 μl of GT-T551 culture medium containing 10% FBS, 5 ng/mL of IL2 and each 0.1 μg of #21-3BiTE, #21-3 or mouse IgG for 4 days. The gated CellTrace-Blue^+^ cells were assessed by FACS.

### *In vitro* T-cell-dependent cellular cytotoxicity assay

T-cells were mixed with tumor cells that stably expressed luciferase at an E:T ratio of 10:1 (1 × 10^5^:1 × 10^4^) in 96-well plates and cultured in GT-T551 culture medium that contained 10% FBS and 5 ng/mL IL2 with 0.01~1 µg/mL antibodies. After the indicated period of incubation, the cells were washed with PBS, and 100 µl of cell lysis solution that contained luciferase substrate was added to each well of the 96-well plates. Bioluminescence was measured for 1 second with a Varioskan LUX multimode reader (Thermo Fisher Scientific) as relative light units (RLU). The assay measures target cell viability by calculating the number of viable luciferase positive cells. The 100% viability reference point (maximal RLU) was determined by plating target cells (T) and effector cells (E) in media without antibody (Ab). Cell viability was calculated from the data with the following equation: cell viability (%)  =  100 × (E + T + Ab)/(maximal RLU). Media RUL values were subtracted from all samples.

### Data analysis

Statistically significant differences (P < 0.001) between groups were determined by ANOVA followed by Tukey’s Multiple Comparison Test or Dunnett’s test. Statistical analysis was performed by using JMP Pro14 software (SAS Institute Inc., Cary, NC, 1989-2019).

### Study Approval

All experiments were performed in accordance to relevant guidelines and regulations. Animal experimental protocols were approved by the Committee on Animal Experimentation at the University of Toyama and conducted using project licence A2017eng-1. This study was approved by the Ethics Committee on University of Toyama and conducted using project licence RinNin21-47.

## Supplementary information

Supplementary figures

## Data Availability

The data that support the findings of this study are available from the corresponding authors upon reasonable request.
