# Supplementary material for: High throughput development of TCR-mimic antibody that targets survivin-2B80-88/HLA-A*A24 and its application in a bispecific T-cell engager
Source: Sci Rep. 2019 Jul 8;9:9827. doi: 10.1038/s41598-019-46198-5 (PMC6614450; doi:10.1038/s41598-019-46198-5)
Supplement: Supplementary file 1 — Supplementary figures [file 41598_2019_46198_MOESM1_ESM.pdf]

High throughput development of TCR-mimic antibody that targets survivin-2B$_{80-88}$/HLA-A*A24 and its application in a bispecific T-cell engager

Nobuyuki Kurosawa[1]*, Yuka Wakata[2], Kenta Ida[3], Aki Midorikawa[3] and Masaharu Isobe[1]*

[1]Laboratory of Molecular and Cellular Biology, Faculty of Science and Engineering, Graduate School, University of Toyama, 3190 Gofuku, Toyama-shi, Toyama, 930-8555, Japan

[2]Frontier Research Core for Life Sciences, University of Toyama, 3190 Gofuku, Toyama-shi, Toyama, 930-8555, Japan

[3]Graduate School of Science and Engineering for Education, University of Toyama, Toyama-shi, Toyama, 930-8555, Japan

Supplemental figures

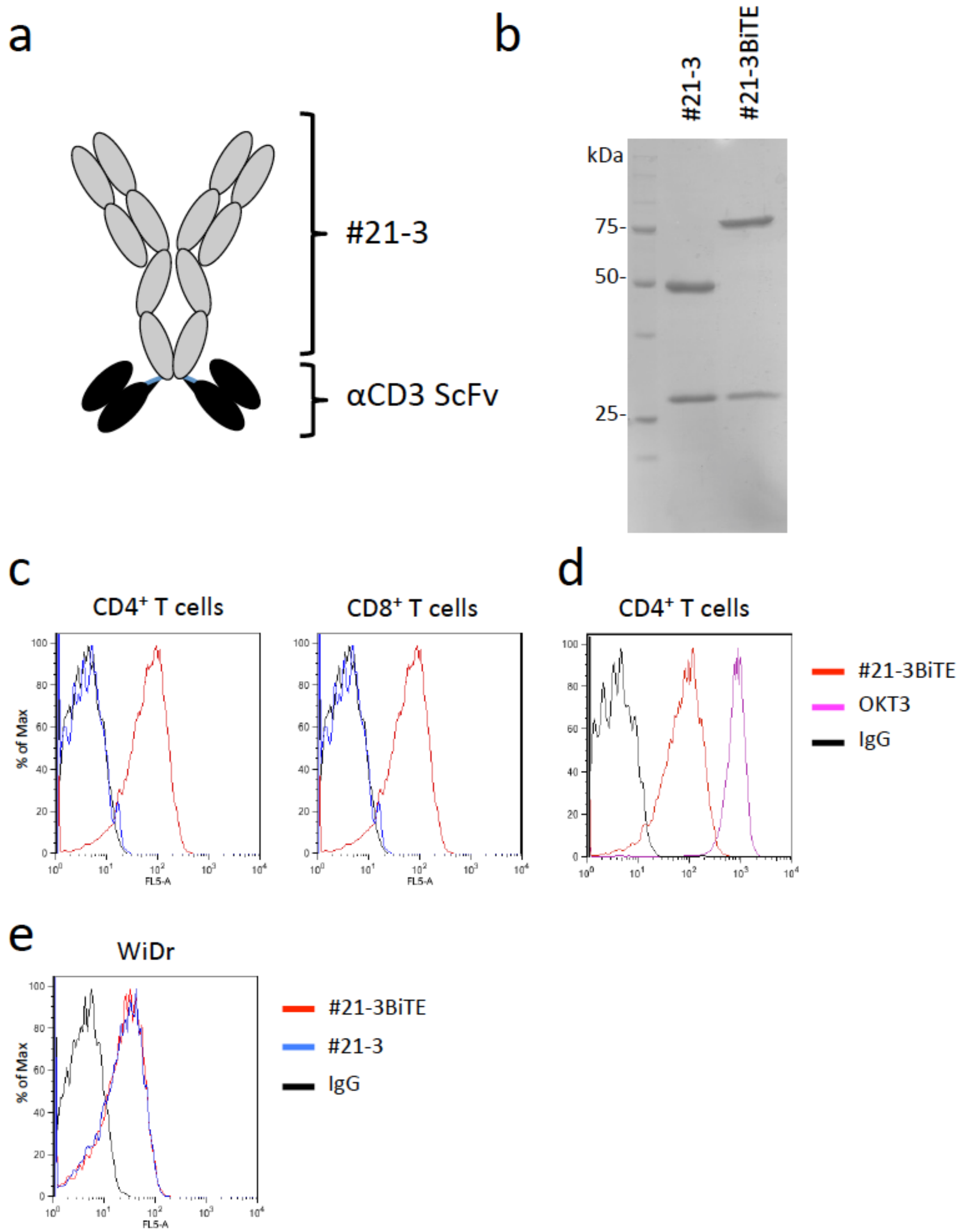

Fig. S1 #21-3BiTE simultaneously binds to human tumor cells and human T-cells.

(a) Schematic diagram of #21-3BiTE.

(b) Reducing SDS-PAGE of the purified #21-3 and #21-3BiTE. Antibodies were separated by SDS–PAGE and stained with Coomassie brilliant blue R. The gel images were acquired with E-BOX-Electrophoresis Gel Photodocumentation System (VILBER, https://www.vilber.com/e-box/).

(c) PBMC from a HLA-A*24^{-} healthy human donor were stained with 0.1 μg/mL of #21-3BiTE, #21-3 or control mouse IgG, followed by addition of antibody against CD4 and CD8, and subsequently analyzed by FACS to detect antibody binding.

(d) CD4^{+} T-cells in (c) were incubated with 0.1 μg/mL of #21-3BiTE, anti CD3 antibody (OKT3) or control mouse IgG, and subsequently analyzed by FACS.

(e) WiDr cells were incubated with #21-3BiTE, #21-3 or control mouse IgG and subsequently analyzed by FACS.

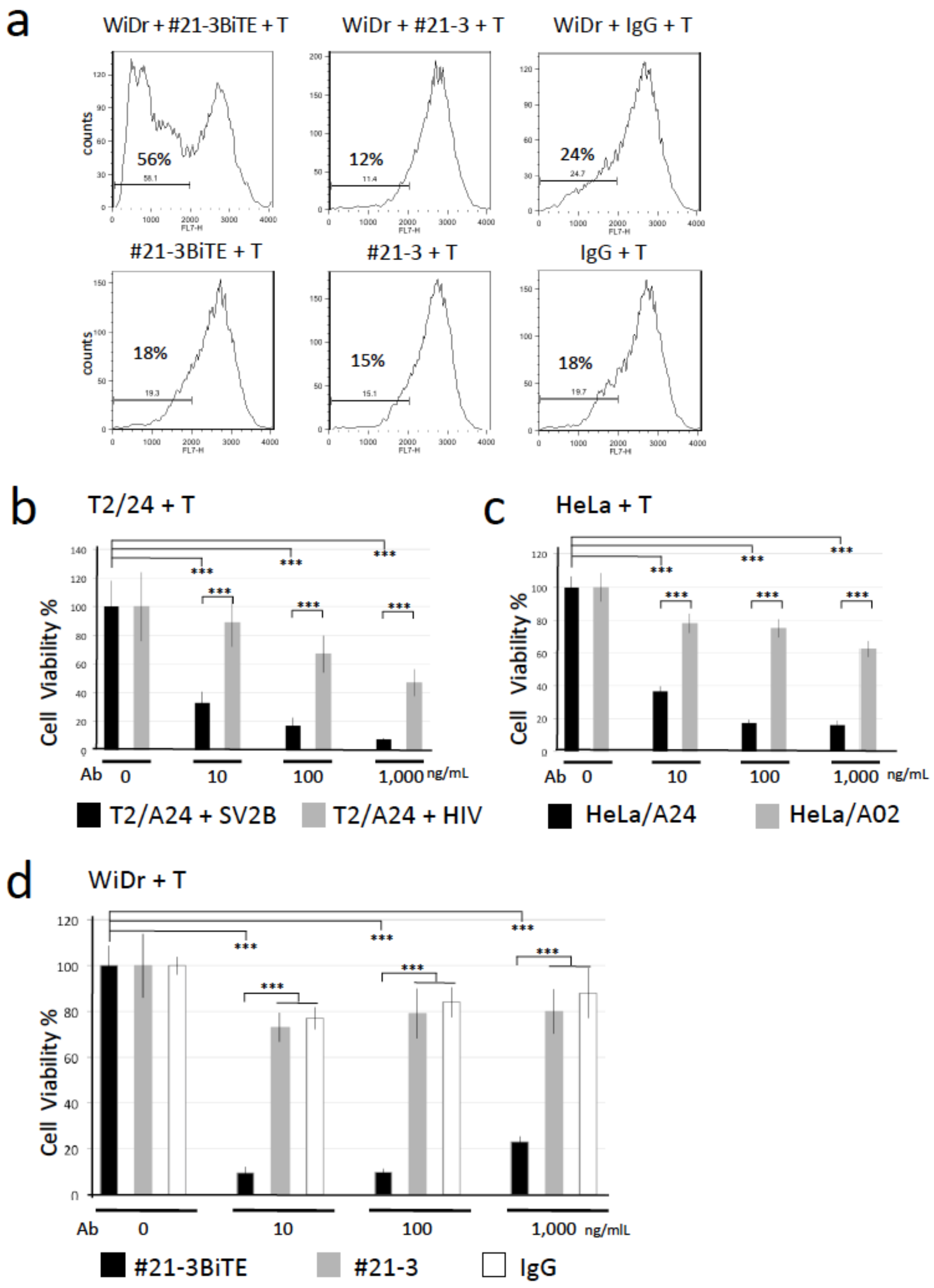

Fig. S2 BiTE-dependent T-cell-mediated cytotoxicity against tumor cells.

(a) CellTrace-Blue-labeled T cells prepared from a HLA-A*24$^-$ healthy donor were mixed with or without WiDr cells at an E:T ratio of 10:1 in the presence of the indicated antibody, and the reactions were allowed to proceed for four days. CellTrace-Blue$^+$ cells analyzed by FACS for proliferation are shown.

(b) T2/A24 cells stably expressing luciferase were pulsed with the indicated peptide and subsequently mixed with T-cells expanded from a HLA-A*24$^-$ healthy donor at an E:T ratio of 10:1 in the presence of serial dilutions of #21-3BiTE. The reactions were allowed to proceed for 8 h.

(c) HeLa/A24 or HeLa/A02 cells stably expressing luciferase were mixed with T-cells expanded from a HLA-A*24$^-$ healthy donor at an E:T ratio of 10:1 in the presence of serial dilutions of #21-3BiTE, and the reactions were allowed to proceed for 12 h.

(d) WiDr-luci cells were treated as in (c), and the reactions were allowed to proceed for 14 h. Target cell viability was determined by luciferase assay. All data are representative of two independent experiments. Relative cell viability values for target cells are represented as the average ± SD for three replicates/group (***$p<0.001$).
